# Angiogenic Factors and Inflammatory Bowel Diseases

**DOI:** 10.3390/biomedicines13051154

**Published:** 2025-05-09

**Authors:** Zhiru Li, Li Zeng, Wei Huang, Xinxing Zhang, Li Zhang, Qin Xie

**Affiliations:** 1 Clinical Medical School, University of Electronic Science and Technology of China, Chengdu 610072, China; lizhiru050525@163.com; 2 Department of Geriatric Medicine, Sichuan Provincial People’s Hospital, University of Electronic Science and Technology of China, Chengdu 610072, China; 13348884951@163.com (L.Z.); huangweihw225@163.com (W.H.); zhangxinxing1979@sohu.com (X.Z.); zl.glx.gjq@163.com (L.Z.)

**Keywords:** inflammatory bowel disease, angiogenesis, angiogenic factors

## Abstract

Inflammatory bowel disease (IBD), including Crohn’s disease and ulcerative colitis, is characterized by chronic intestinal inflammation and impaired epithelial barrier function. Emerging evidence highlights the critical role of vascular remodeling and angiogenesis in IBD pathogenesis. This review explores the intricate relationship between blood vessels and the intestinal epithelial barrier, emphasizing how aberrant vascularization contributes to barrier dysfunction and disease progression. In IBD, excessive angiogenesis is driven by hypoxia, immune cell infiltration, and pro-inflammatory cytokines, further perpetuating inflammation and tissue damage. Key angiogenic factors, such as vascular endothelial growth factor (VEGF), angiopoietins, and platelet-derived growth factor (PDGF), are upregulated in IBD, promoting pathological vessel formation. These newly formed vessels are often immature and hyperpermeable, exacerbating leukocyte recruitment and inflammatory responses. Given the pivotal role of angiogenesis in IBD, anti-angiogenic therapies have emerged as a potential therapeutic strategy. Preclinical and clinical studies targeting VEGF and other angiogenic pathways have shown promise in reducing inflammation and promoting mucosal healing. This review summarizes current knowledge on vascular–epithelial interactions in IBD, the mechanisms driving pathological angiogenesis, and the therapeutic potential of anti-angiogenic approaches, providing insights for future research and treatment development.

## 1. Introduction

Inflammatory Bowel Disease (IBD) is a chronic, recurrent inflammatory disorder of the intestine, primarily encompassing Crohn’s disease (CD) and ulcerative colitis (UC). In recent years, with shifts in lifestyle and environmental factors, the incidence of IBD has been steadily increasing globally [1,2], posing a significant challenge to public health systems worldwide. IBD not only severely impacts patients’ quality of life through symptoms such as abdominal pain, diarrhea, and weight loss, but also imposes a considerable economic burden on healthcare systems due to the costs associated with diagnosis, treatment, and management [3,4,5].

Despite extensive research endeavors, the precise etiology and pathogenesis of IBD remain elusive. However, it is widely acknowledged that a complex interplay between genetic predisposition, immune dysfunction, environmental triggers, and alterations in gut microbiota plays crucial roles in disease onset and progression [6,7,8,9]. One emerging area of scientific interest is the role of angiogenesis in the pathogenesis of IBD.

Angiogenesis, the physiological process of forming new blood vessels from pre-existing vasculature, is vital for normal growth and development. However, it also plays a pivotal role in numerous disease states, including cancer and inflammatory conditions [10]. In the context of IBD, angiogenesis is believed to contribute to the chronic inflammatory process by facilitating the influx of immune cells and inflammatory mediators into the intestinal mucosa [11,12,13,14]. This process leads to the destruction of the intestinal barrier, further exacerbating inflammation and tissue damage.

## 2. The Vascular and Epithelial Barrier

The vascular and epithelial barrier forms a critical interface between the intestinal lumen and the underlying tissue, regulating the influx of nutrients, waste products, and immune cells [15,16]. This intricate barrier system ensures the selective permeability necessary for maintaining gut homeostasis. In inflammatory bowel diseases (IBD), this barrier function is compromised, leading to increased permeability and chronic inflammation [17,18].

The vascular endothelium plays a central role in maintaining barrier integrity [19,20,21]. Endothelial cells line the interior surface of blood vessels and regulate the passage of cells and molecules between the blood and tissues through tight junctions and adherens junctions [22]. In IBD, endothelial cells undergo functional and structural changes, including increased permeability, adhesion molecule expression (such as ICAM-1 and VCAM-1), and leukocyte recruitment [23,24]. These changes are orchestrated by a complex interplay of inflammatory cytokines (e.g., TNF-α, IL-1β, and IL-6), chemokines (e.g., CXCL8/IL-8), and reactive oxygen species (ROS), which collectively contribute to a breakdown of barrier function and persistent inflammation [25,26]. For instance, TNF-α has been shown to disrupt endothelial tight junctions and enhance vascular permeability in animal models of IBD [27].

Similarly, the epithelial barrier, composed of a single layer of intestinal epithelial cells (IECs), is crucial for maintaining gut homeostasis [28,29,30]. IECs form a tight, selective barrier through tight junctions and adherens junctions, preventing the entry of luminal antigens and pathogens into the mucosa [31]. In IBD, epithelial cells undergo apoptosis, proliferation, and differentiation abnormalities, disrupting barrier function and facilitating the entry of luminal antigens and bacteria into the mucosa [32,33]. This disruption triggers an immune response, characterized by the activation of innate and adaptive immune cells, leading to chronic inflammation and tissue damage [34].

**Figure 1:**
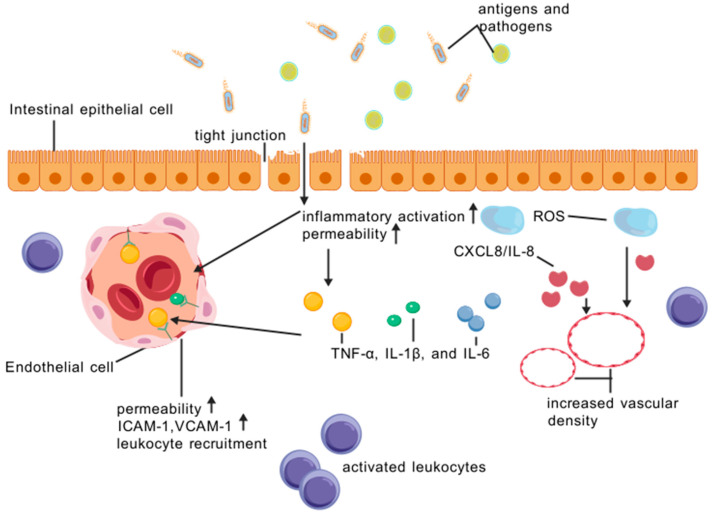
Intestinal epithelial cells maintain the integrity of the barrier through tight junctions. When encountering antigens and pathogens, the tight junction was destroyed and intestinal epithelial cells trigger an inflammatory activation response, producing a series of inflammatory mediators such as ROS reactive oxygen species, TNF-α, IL-1β, and IL-6. At the same time, the release of chemokine CXCL8/IL-8 guides the movement of white blood cells to the infection site, further promoting the inflammatory response. In IBD, inflammation activates the intestinal microvasculature and endothelial cells undergo functional and structural changes, manifested by increased permeability, upregulation of adhesion molecules such as ICAM-1 and VCAM-1, and increased recruitment of leukocytes. These changes are coordinated by complex signaling pathways, leading to increased vascular permeability and easier entry of white blood cells through the vascular wall into tissues, thereby exacerbating inflammatory reactions.

The interplay between the vascular and epithelial barrier in IBD is complex and bidirectional [13,35]. Increased vascular permeability allows for the influx of inflammatory cells and mediators (such as cytokines, chemokines, and ROS) into the tissue, exacerbating the inflammatory response [36]. Conversely, epithelial barrier dysfunction facilitates the entry of luminal antigens and bacteria into the lamina propria, perpetuating the inflammatory cycle through the activation of pattern recognition receptors (e.g., TLRs) on immune cells [37]. This vicious cycle of inflammation and barrier dysfunction contributes to the chronicity and relapsing-remitting nature of IBD (Figure 1).

Understanding the mechanisms underlying barrier dysfunction is crucial for developing effective therapies to restore gut homeostasis in IBD. Various therapeutic strategies, including anti-inflammatory drugs, biologics targeting cytokines (e.g., TNF-α inhibitors), and probiotics, have shown promise in improving barrier function and reducing inflammation in IBD [38,39]. Additionally, emerging research on the gut microbiota, epithelial cell regeneration, and targeting tight junction proteins offers new avenues for therapeutic intervention [20,21,29,30,40,41,42,43,44,45,46]. By elucidating the intricate interplay between the vascular and epithelial barrier in IBD, researchers can continue to advance our understanding of this complex disease and develop more effective treatments to improve patient outcomes.

## 3. Mechanism of Angiogenesis in IBD

An important aspect of IBD pathology involves changes in the vasculature, where endothelial cell activation and remodeling, as well as intussusceptive angiogenesis (IA), play crucial roles in the progression and maintenance of IBD inflammation [14,47].

In IBD, the density of microvasculature is significantly increased in the inflamed mucosa, correlating directly with disease severity. This increased vascular density is driven by the release of pro-angiogenic factors such as VEGF (vascular endothelial growth factor), bFGF (basic fibroblast growth factor), and PDGF (platelet-derived growth factor) [14,48,49,50,51,52,53]. The new vessels formed during IBD are often immature and leaky. They lack proper pericyte coverage, leading to increased vascular permeability and edema. This contributes to the overall inflammatory environment and tissue damage [54,55].

Angiogenesis can be categorized into two primary mechanisms: extension from existing vessels (sprouting angiogenesis) and vessel splitting (intussusceptive angiogenesis). Both mechanisms play significant roles in the pathogenesis and progression of IBD.

### 3.1. Sprouting Angiogenesis

Sprouting angiogenesis involves the extension of new blood vessels from pre-existing ones. This process is particularly prominent in inflamed tissues, where hypoxia—a common feature—plays a crucial role. Hypoxia induces the expression of vascular endothelial growth factor (VEGF) through the activation of hypoxia-inducible factors (HIF-1α and HIF-2α) [56]. VEGF is a potent stimulator of angiogenesis and acts by binding to its receptors (VEGFR-1, VEGFR-2) on endothelial cells, initiating a cascade of events that lead to the formation of new vessels.

The process of sprouting angiogenesis begins with the degradation of the basement membrane by matrix metalloproteinases (MMPs), allowing endothelial cells to migrate and proliferate. These cells form new capillary sprouts that extend towards the source of VEGF. The formation of new vessels is further supported by the recruitment of pericytes and smooth muscle cells, which stabilize the newly formed vessels. However, in IBD, the newly formed vessels are often immature and leaky, lacking proper pericyte coverage, which leads to increased vascular permeability and edema [54]. This increased permeability allows more immune cells and inflammatory mediators to reach the inflamed tissue, perpetuating the inflammatory cycle.

### 3.2. Intussusceptive Angiogenesis

Intussusceptive angiogenesis, also known as splitting angiogenesis, involves the splitting of existing vessels through intraluminal endothelial cell rearrangements. This process is regulated by several factors, including nitric oxide (NO), endoglin, and the ephrinB2/EphB4 signaling pathways [57,58,59]. Unlike sprouting angiogenesis, which involves the proliferation of endothelial cells, intussusceptive angiogenesis occurs through the rearrangement of existing endothelial cells within the vessel lumen, creating new vessels without significant cell proliferation.

In IBD, intussusceptive angiogenesis has been observed in murine models of colitis and is thought to contribute significantly to vascular remodeling [57,60,61]. This form of angiogenesis is particularly relevant in the context of IBD because it can occur rapidly in response to changes in blood flow and mechanical forces within the inflamed tissue. The process is initiated by the formation of a pillar within the lumen of the vessel, which then splits the vessel into two new vessels. This splitting process is regulated by the balance of pro-angiogenic and anti-angiogenic factors, with NO playing a crucial role in promoting intussusceptive angiogenesis [60].

The ephrinB2/EphB4 signaling pathway is also essential for the regulation of intussusceptive angiogenesis. EphB4 is a receptor tyrosine kinase that interacts with its ligand ephrinB2 to control the rearrangement of endothelial cells [59]. This signaling pathway ensures that the process of vessel splitting is tightly regulated and coordinated, preventing excessive vascular permeability and maintaining vascular integrity.

## 4. Inflammation and Angiogenesis in IBD

In the healthy intestinal mucosa, particularly the colon, the mucosal microvasculature plays a pivotal role in maintaining physiological homeostasis. Beyond facilitating the absorption of essential nutrients, electrolytes, and water, it acts as a dynamic barrier that limits microbial translocation from the luminal space while enabling immune surveillance. This equilibrium is disrupted in inflammatory bowel diseases (IBD), where chronic inflammation triggers profound alterations in the intestinal microvasculature, driving a vicious cycle that perpetuates tissue damage and disease progression [50].

During IBD, immune cells infiltrate the intestinal mucosa and secrete pro-inflammatory cytokines (e.g., TNF-α, IL-1β) and growth factors (e.g., VEGF, FGF), which directly activate endothelial cells (ECs). This activation induces the expression of adhesion molecules (e.g., ICAM-1, VCAM-1) on EC surfaces, promoting leukocyte recruitment and extravasation—hallmarks of inflammation. Simultaneously, the inflammatory milieu disrupts vascular integrity, leading to hyperpermeability and edema. Concurrently, angiogenesis is upregulated through two distinct mechanisms: sprouting angiogenesis (via EC proliferation and tube formation) and intussusceptive angiogenesis (via vascular splitting and remodeling) [62,63]. These processes collectively expand the microvascular network to meet the metabolic demands of the inflamed tissue.

However, this adaptive response becomes maladaptive in chronic inflammation. Hypoxia, a consequence of oxygen consumption by infiltrating immune cells and damaged epithelial cells, further stimulates angiogenesis by recruiting macrophages and other inflammatory cells to hypoxic regions [48]. These cells secrete angiogenic factors, including proteases (e.g., MMPs) and nitric oxide (NO), which degrade the extracellular matrix and promote EC migration. The newly formed vessels, in turn, exacerbate inflammation by delivering oxygen, nutrients, and additional inflammatory mediators to the site, sustaining immune cell recruitment and cytokine production. This reciprocal stimulation between inflammation and angiogenesis creates a self-reinforcing loop that amplifies tissue injury, fibrosis, and barrier dysfunction.

Clinically, this interplay underscores the challenges in treating IBD, as therapies targeting either process alone often fail to break the cycle. For instance, anti-VEGF agents may reduce vascularization but inadvertently worsen hypoxia, triggering compensatory inflammatory pathways [64]. Conversely, anti-inflammatory drugs may mitigate cytokine storm but leave residual angiogenesis to fuel disease relapse. A holistic approach disrupting both axes—e.g., by targeting hypoxia-inducible factors (HIFs) or macrophage polarization—may offer more durable benefits.

## 5. Angiogenic Growth Factors in IBD

A diverse array of angiogenic mediators play pivotal roles in the pathogenesis of Inflammatory Bowel Disease (IBD), including Vascular Endothelial Growth Factor (VEGF), Platelet-Derived Growth Factor (PDGF), Fibroblast Growth Factors (FGFs), angiopoietins, Matrix Metalloproteinases (MMPs), and several other factors. These mediators are integral in regulating angiogenesis, vascular permeability, and inflammatory cell recruitment, thereby contributing to the complex inflammatory processes observed in IBD.

### 5.1. Vascular Endothelial Growth Factor (VEGF)

The VEGF family encompasses VEGF-A, VEGF-B, VEGF-C, VEGF-D, VEGF-E, as well as placental growth factor [65]. Notably, VEGF-A was the inaugural member of this family to be discovered and remains the most extensively researched to date. During inflammatory processes, a range of cells, including stromal cells, neutrophils, monocytes, and endothelial cells themselves, can produce VEGF-A. Research has demonstrated a connection between VEGF-A and VEGF-B with angiogenesis [66,67,68]. VEGF-A elicits transient vasodilatation and augments vascular permeability via the liberation of nitric oxide (NO), thereby stimulating the proliferation, directional migration, and differentiation of vascular endothelial cells. It plays a crucial role in both physiological growth and pathological angiogenesis.

Scaldaferri et al. [66] found that in mice with dextran sulfate sodium (DSS)-induced colitis, overexpression of VEGF-A exacerbated the disease condition by enhancing intestinal mucosal angiogenesis and stimulating leukocyte adhesion in vivo. Conversely, inhibiting VEGF-A expression was significantly effective in improving intestinal symptoms in these mice, while also suppressing the production of mucosal inflammatory cytokines. Elevated levels of VEGF-A and VEGFR-2 were observed in intestinal samples from both IBD patients and DSS-induced colitis mice. Another study also found that VEGF levels were significantly upregulated in the mucosa of intestinal inflammatory lesions in patients with CD and UC [49,69], indicating that VEGF and angiogenesis play crucial roles in the pathogenesis of IBD. Danese et al. [47] reported increased microvascular density in the intestinal mucosa of IBD patients, with upregulated expression of VEGF, TNF-α, bFGF, and IL-8 in human intestinal microvascular endothelial cells (HIMECs). Furthermore, the intestinal mucosa of IBD patients effectively induced neovascularization in the cornea and chick chorioallantoic membrane. These findings robustly demonstrated active neovascularization in IBD from three aspects: intestinal mucosal neovascular morphology, angiogenic marker expression, and neovascular function. Since multiple studies have shown that pathological angiogenesis is a critical aspect of the onset and progression of IBD, researchers proposed the hypothesis that the anti-angiogenic factor endostatin should exert a protective effect on IBD. This hypothesis was confirmed in three experimental animal models: iodoacetamide-induced mice, DSS-induced mice, and IL-10 gene knockout mice [70].

Another study demonstrated that VEGF-C-dependent stimulation of lymphatic function ameliorates experimental IBD, suggesting a protective mechanism through enhanced lymphatic drainage [71]. Conversely, Wang et al. reported that promoting inflammatory lymphangiogenesis by VEGF-C aggravated intestinal inflammation in mice with experimental acute colitis, highlighting a context-dependent pro-inflammatory effect [72]. Additionally, Wang et al. found that exosomes from adipose-derived stem cells promote VEGF-C-dependent lymphangiogenesis by regulating the miRNA-132/TGF-beta pathway, providing a potential therapeutic avenue via stem cell-derived exosomes [73]. These studies collectively illustrate the complex interplay of VEGF-C in IBD, where its effects can be both protective and pathogenic, dependent on the specific disease context and regulatory pathways involved.

A meta-analysis examining subtypes of inflammatory bowel disease (IBD) has shed light on the distinct pathophysiological features between ulcerative colitis (UC) and Crohn’s disease (CD) through the lens of vascular endothelial growth factor (VEGF) concentrations [48]. The study revealed notably higher VEGF levels in UC patients compared to those with CD. This discrepancy in VEGF concentrations likely mirrors the fundamental differences in the inflammatory and angiogenic processes underlying these two conditions.

UC predominantly manifests as a continuous mucosal inflammation that typically initiates in the rectum and spreads proximally along the colon. This type of inflammation is largely confined to the mucosal layer. In contrast, CD is characterized by transmural inflammation, which means it can penetrate through the entire thickness of the bowel wall. Moreover, CD has a more unpredictable pattern of involvement, as it can affect any segment of the gastrointestinal tract, from the mouth to the anus [74,75,76].

Elevated levels of VEGF play a pivotal role in stimulating endothelial cell proliferation, migration, and tube formation [66,67,68]. This intricate process is crucial for the formation of new blood vessels, a phenomenon that can exacerbate inflammation by increasing the vascular supply to already inflamed tissues. The augmented blood supply not only feeds the inflammatory process but also facilitates the delivery of immune cells and inflammatory mediators to the site of disease, perpetuating tissue damage [66,77,78].

Moreover, VEGF exhibits a profound impact on vascular permeability. By enhancing the permeability of blood vessels, VEGF allows for the influx of inflammatory cells and mediators into the intestinal mucosa. This influx further amplifies the inflammatory cascade, contributing to the chronic and relapsing nature of IBD [77,78].

As a member of the VEGF family, placental growth factor (PlGF) is produced and secreted by various stromal cells in vivo. Nejabati et al. explored the dual role of PlGF as an angiogenic and inflammatory switcher, drawing lessons from early pregnancy losses. While the primary focus of this study was not IBD, it highlighted PlGF’s ability to regulate angiogenesis and inflammation [79]. Zhou et al. specifically investigated the role of PlGF in enhancing angiogenesis in human intestinal microvascular endothelial cells via the PI3K/Akt pathway. Their findings directly implicate PlGF in the regulation of angiogenesis in the intestinal microvasculature, which is particularly relevant to IBD. By stimulating the PI3K/Akt pathway, PlGF promotes the proliferation, migration, and tube formation of endothelial cells, all critical steps in angiogenesis [80], which suggests that PlGF could be a therapeutic target for modulating angiogenesis and potentially alleviating IBD symptoms.

### 5.2. Angiopoietins (ANG)

Angiopoietins are a class of angiogenic factors, with four family members identified so far: Ang-1, Ang-2, Ang-3, and Ang-4 [81]. Among them, Ang-1 exhibits protective effects on blood vessels and inhibits inflammation by recruiting peri-endothelial cells to maintain vascular integrity. In contrast, Ang-2 disrupts the balance of vascular growth and plays a significant role in vascular remodeling and pathological angiogenesis. Both Ang-1 and Ang-2 bind to the tyrosine kinase receptor Tie-2 to regulate vascular stability signals, with Ang-2 serving as a natural antagonist to Ang-1. Oikonomou et al. [82] conducted a study analyzing 52 UC patients, 59 CD patients, and 55 healthy controls. They found statistically significant differences in angiopoietin levels between UC and CD patients compared to the control group, with lower Ang-1 levels and higher Ang-2 levels in the patient groups. Notably, CD patients with disease limited to the colon had lower Ang-2 levels than other patients. Another study also confirmed that serum Ang-2 levels were higher in UC and CD patients compared to healthy controls [83,84]. Mice with Ang-2 knockout exhibited reduced intestinal inflammatory symptoms, decreased neutrophil infiltration, and reduced angiogenesis after DSS induction [83].

### 5.3. Platelet-Derived Growth Factor (PDGF)

PDGF is known to be elevated in the serum and tissues of IBD patients, particularly during active phases of the disease. This elevation is associated with increased angiogenesis, fibrosis, and tissue remodeling, which are common features of chronic inflammation in IBD. Specifically, PDGF-BB, one of the isoforms of PDGF, has been shown to correlate strongly with clinical features of IBD, including endoscopic activity and disease severity [85,86,87]. Recent studies have highlighted the importance of PDGF in predicting and modulating treatment response in IBD. For instance, a pilot study aimed to identify biological differences associated with differential treatment response to Vedolizumab, an anti-α4β7 integrin therapy. The study measured a broad range of analytes in the serum of patients before and after treatment initiation. It was found that responders to Vedolizumab had higher baseline levels of PDGF-ββ compared to non-responders [88]. This suggests that PDGF levels could serve as a potential biomarker for predicting treatment response to Vedolizumab.

### 5.4. Basic Fibroblast Growth Factors (FGFs)

bFGF, also known as Fibroblast Growth Factor-2 (FGF-2), is a heparin-binding protein and one of the earliest discovered members of the FGF family. It is expressed in fibroblasts, smooth muscle cells, and endothelial cells. bFGF stimulates the proliferation of vascular endothelial cells, induces the production of plasminogen activators, increases collagenase activity, and exhibits potent angiogenic effects. Studies by Di Sabatino et al. [89] and Danese et al. [47] both found increased levels of bFGF and VEGF in CD patients. However, another study on pediatric CD patients showed no significant difference in bFGF levels compared to other control groups, but there was a correlation between bFGF expression levels and disease activity [90].

### 5.5. Inducible Nitric Oxide Synthase (iNOS)

Among the myriads of mediators that regulate microvascular function, nitric oxide (NO) has garnered significant attention due to its diverse and pivotal roles. Under physiological conditions, NO functions to counteract leukocyte and platelet adhesion to endothelial cells (ECs), regulate vasodilatation and endothelial permeability, and act as a radical scavenger [91]. In the context of IBD, the intricate balance of NO production and its effects on vascular function become particularly relevant.

In healthy intestinal microvascular endothelial cells (HIMECs), the expression of both endothelial nitric oxide synthase (eNOS) and inducible nitric oxide synthase (iNOS) indicates a high tolerance towards inflammatory activation [92]. Under normal circumstances, the resultant high levels of NO inhibit the expression of endothelial cell adhesion molecules (CAMs) and matrix metalloproteinases (MMPs) induced by inflammatory cues (ICs), thereby maintaining vascular homeostasis.

However, during IBD, the production of NO by ECs is compromised due to the loss of iNOS and eNOS in HIMECs, leading to increased leukocyte adhesion [93,94]. Furthermore, an upregulation of arginase expression and activity has been observed in inflamed HIMECs. Arginase competes with NOS for the substrate L-arginine, thus limiting NO production due to reduced substrate availability [94]. This decreased production of NO in ECs results in the loss of NO-mediated vasodilatation and an increase in reactive oxygen species (ROS) production in the microvessels of affected intestinal areas [95,96].

NO also plays a crucial role in angiogenesis, specifically in VEGF-driven angiogenesis and intussusceptive angiogenesis [61]. In IBD, the reduced NO levels may impair these angiogenic processes, contributing to both the perpetuation of inflammation and impaired wound healing. Additionally, NO has been shown to regulate endothelial barrier function in human, murine, and bovine ECs by promoting VEGF-induced permeability through targeting the VE-cadherin/β-catenin and Rho pathways [97,98]. However, NO can also protect ECs from hypoxia-induced barrier dysfunction [99], highlighting its complex and context-dependent roles.

The deficiency of eNOS and iNOS in mouse models of colitis has been associated with varying disease outcomes, ranging from a better to a more severe course of disease [100,101,102,103]. These differences might be attributed to the distinct roles played by NO in different cell compartments and under different conditions. For instance, the expression of eNOS by intestinal endothelial cells has been shown to specifically maintain mucosal integrity and prevent bacterial translocation in a TNBS-colitis model in mice [103].

### 5.6. Other Factors Related to Angiogenesis in IBD

Previous studies showed that cytokines (e.g., TNF-α, IFN-γ, and IL-1β) could affect the angiogenesis in IBD, in which TNF-α was the most important [104,105].

In intestinal inflammation, TNF-α is known to induce a cascade of inflammatory responses, leading to the recruitment of various immune cells and the production of additional inflammatory cytokines. This inflammatory milieu can promote angiogenesis, as evidenced by studies showing enhanced platelet adhesion and subsequent angiogenesis in intestinal inflammation and IBD microvasculature [106]. However, the net effect of TNF-α on angiogenesis in IBD is often inhibitory, particularly when anti-TNF-α therapies are administered [107].

Anti-TNF-α antibody therapy, such as infliximab, has been shown to support the recovery of eNOS (endothelial nitric oxide synthase) and VEGFR2 (vascular endothelial growth factor receptor 2) protein expression in endothelial cells. These proteins are crucial for angiogenesis, as they regulate endothelial cell proliferation, migration, and survival. By inhibiting TNF-α, infliximab therapy can indirectly promote the restoration of normal angiogenic processes disrupted by inflammation [108].

Matrix metalloproteinases (MMPs) are a class of enzymes that play a key role in tissue remodeling and inflammatory responses [109,110]. Among them, MT1-MMP (matrix metalloproteinase-14) demonstrates important biological functions in IBD. MT1-MMP not only participates in matrix degradation but also affects the function of immune cells by regulating cell signaling. For example, it can promote the migration and activation of macrophages, thereby enhancing local immune responses. Studies have shown that in IBD patients, MT1-MMP promotes the binding of its C-terminal fragment to αvβ3 integrin by cleaving matrix protein TSP1, thereby inducing nitric oxide (NO) production and vasodilation, initiating the IA process, degrading the extracellular matrix to promote neovascularization, and further enhancing local inflammation [61]. In mouse models, endothelial cells lacking MT1-MMP exhibit limited IA, resulting in better tissue perfusion, preserved intestinal morphology, and a lower disease activity index, indicating that MT1-MMP is a potential therapeutic target for IBD. Inhibitory antibodies or non-peptide fragments targeting MT1-MMP can reduce IA in mouse colitis.

Understanding the intricate roles of these angiogenic mediators in IBD is essential for the development of targeted therapies aimed at inhibiting angiogenesis and reducing inflammation. However, the complex interplay between different angiogenic pathways and mediators in IBD presents significant challenges for therapeutic development, necessitating further research to elucidate the underlying mechanisms and identify novel therapeutic targets.

## 6. Antiangiogenic Therapies in IBD

Given the crucial role of angiogenesis in IBD pathogenesis, antiangiogenic therapies have emerged as promising treatment options. These therapies target different angiogenic pathways and mediators to inhibit angiogenesis and reduce inflammation.

VEGF inhibitors constitute the most extensively studied class of antiangiogenic therapies in inflammatory bowel disease (IBD). Bevacizumab, a monoclonal antibody directed against VEGF-A, has demonstrated encouraging results in mitigating inflammation and inducing remission in patients with ulcerative colitis (UC) and Crohn’s disease (CD) [111,112,113]. However, bevacizumab’s therapeutic journey is not devoid of adverse effects, which include gastrointestinal perforation, infection, and hypertension [111,113,114]. These side effects necessitate careful consideration and monitoring in clinical practice.

Other VEGF inhibitors, such as aflibercept and ramucirumab, are also actively being explored for the treatment of IBD. Aflibercept, a fusion protein that binds VEGF-A, VEGF-B, and placental growth factor (PlGF), has shown potential in preclinical studies, although clinical trial data in IBD are still forthcoming [115]. Similarly, ramucirumab, a monoclonal antibody targeting VEGF receptor-2, is under investigation with the hope of offering an alternative to existing VEGF-directed therapies, albeit with its own safety profile to be established [116].

Parallel to VEGF inhibitors, PDGF inhibitors represent another class of antiangiogenic therapies in IBD. Imatinib, a tyrosine kinase inhibitor targeting PDGF receptor signaling, has shown promise in reducing inflammation and tissue damage in animal models of IBD [117]. Unfortunately, imatinib’s clinical application is hindered by adverse effects such as nausea, vomiting, and myelosuppression [118], which limit its broader use in IBD patients.

In addition, there are some treatments for inflammatory bowel disease that target cytokines, such as Guselkumab, a humanized IgG1 monoclonal antibody that can specifically bind to the p19 subunit of IL-23, thereby inhibiting its inflammatory signaling. Guselkumab has previously demonstrated good efficacy and safety in the treatment of psoriasis and Crohn’s disease [119].

Beyond VEGF and PDGF, other antiangiogenic therapies, such as MMP (matrix metalloproteinase) inhibitors and endothelial cell-targeting therapies are in the nascent stages of development for IBD treatment. MMP inhibitors aim to regulate extracellular matrix degradation, a crucial aspect of angiogenesis and inflammation [120]. Endothelial cell-targeting therapies, including those that disrupt specific endothelial cell signaling pathways, hold promise but require further preclinical and clinical validation (Table 1) [121,122].

Despite the encouraging potential of antiangiogenic therapies in IBD, several challenges persist. The intricate interplay among various angiogenic pathways and mediators in IBD complicates therapeutic development, necessitating a nuanced understanding of these interactions. Moreover, the adverse effects associated with current antiangiogenic therapies pose significant limitations to their clinical utility [50,123].

Given these challenges, further research is imperative to identify more specific and effective antiangiogenic therapies for IBD. Advances in precision medicine, including the use of biomarkers to identify patients most likely to respond to specific therapies and those at risk of adverse events, could pave the way for more tailored treatment approaches [63]. Additionally, the exploration of combination therapies targeting multiple angiogenic pathways may enhance therapeutic efficacy while minimizing adverse effects.

In conclusion, while antiangiogenic therapies offer promising avenues for IBD treatment, ongoing research is crucial to overcome existing challenges, optimize therapeutic strategies, and ultimately improve patient outcomes.

## 7. Other Therapies Modulating Angiogenesis in IBD

Antiangiogenic therapies aim to inhibit angiogenesis and reduce inflammation by targeting various angiogenic pathways and mediators. One of the most extensively studied classes of anti-inflammatory therapies in IBD is the anti-TNF-α agents, which indirectly impact angiogenesis by modulating the immune response and reducing cytokine-driven inflammation.

Anti-TNF-α therapies, such as infliximab, adalimumab, and golimumab, are well-established treatments for IBD, particularly for Crohn’s disease (CD) and ulcerative colitis (UC). Their mechanism of action involves neutralizing TNF-α, a cytokine that plays a pivotal role in the inflammatory cascade and promotes angiogenesis through the activation of endothelial cells and the induction of VEGF production [124]. By blocking TNF-α, these therapies can decrease vascular permeability, inhibit endothelial cell proliferation, and reduce the formation of new blood vessels, thereby mitigating inflammation and tissue damage [107,108].

Clinical studies have demonstrated the efficacy of anti-TNF-α therapies in inducing and maintaining clinical remission in patients with IBD [125,126]. However, a subset of patients does not respond to these therapies, and some develop adverse effects, such as infections, infusion reactions, and autoimmune phenomena. Furthermore, the long-term use of anti-TNF-α agents may be associated with the development of anti-drug antibodies, which can lead to loss of response and increased disease activity [127].

In addition to their direct anti-inflammatory effects, anti-TNF-α therapies may also indirectly inhibit angiogenesis by modulating the expression of angiogenic factors. For instance, TNF-α inhibition has been shown to downregulate VEGF and PDGF expression in intestinal tissues of IBD patients, suggesting a potential antiangiogenic mechanism of action [107,108]. However, the precise role of anti-TNF-α therapies in regulating angiogenesis in IBD remains an area of active research.

Despite the challenges and limitations, anti-TNF-α therapies remain a cornerstone of IBD treatment. Future research should focus on identifying biomarkers that can predict response to therapy and developing more targeted antiangiogenic therapies that minimize adverse effects and improve patient outcomes.

## 8. Conclusions

Angiogenesis, the formation of new blood vessels, has long been a topic of significant interest in the context of inflammatory bowel disease (IBD). However, unraveling its precise role in IBD pathogenesis remains a formidable challenge.

Indeed, the intricate relationship between angiogenesis and inflammation in inflammatory bowel disease (IBD) presents a significant challenge, making it difficult to unequivocally determine whether angiogenesis serves as a cause, a propeller, or merely a consequence of the inflammatory processes involved. This complexity underscores the importance of moderating our conclusions and delving deeper into the implications of a more nuanced understanding of angiogenesis in the context of IBD.

In recent years, accumulating evidence has suggested that angiogenesis, the process of new blood vessel formation, plays a pivotal role in the pathogenesis of IBD. On one hand, the influx of inflammatory cells and the subsequent release of pro-inflammatory cytokines necessitate an increased blood supply to sustain the inflammatory response, thereby promoting angiogenesis. On the other hand, the newly formed blood vessels can exacerbate the inflammatory milieu by facilitating the entry of more immune cells and the dissemination of inflammatory mediators. This bidirectional relationship creates a vicious cycle that perpetuates IBD.

Given this interplay, it is imperative to explore the potential therapeutic implications of targeting angiogenesis in IBD. While traditional treatments primarily focus on alleviating inflammation, incorporating anti-angiogenic strategies may offer a novel approach to disrupting this inflammatory–angiogenic axis. Anti-angiogenic factors, such as those that inhibit vascular endothelial growth factor (VEGF) or angiogenin, could potentially mitigate the disease progression by reducing the vascularization of the inflamed tissue.

Moreover, the combination of anti-angiogenic factors with anti-inflammatory drugs merits investigation. By targeting both angiogenesis and inflammation concurrently, such combination therapies may achieve synergistic effects, leading to more pronounced improvements in clinical outcomes. This multifaceted approach recognizes the intertwined nature of the inflammatory and angiogenic processes in IBD and aims to tackle them simultaneously.

However, it is crucial to approach these therapeutic strategies with caution. Further research is needed to elucidate the precise mechanisms underlying the angiogenesis–inflammation interplay in IBD and to identify the optimal targets and timing for anti-angiogenic interventions. Additionally, the potential side effects and long-term safety of anti-angiogenic therapies must be thoroughly evaluated to ensure their safe and effective use in IBD patients.

In conclusion, a better understanding of the role of angiogenesis in IBD holds promise for the development of novel therapeutic strategies. By moderating our conclusions and refining our approach, we can pave the way for more targeted and effective treatments that address both the inflammatory and angiogenic aspects of this complex disease.

## Figures and Tables

**Table 1:** Therapies modulating angiogenesis in IBD.

Drug Type	Drug Name	Target	Side Effects
VEGF Inhibitor	Bevacizumab	VEGF-A	Gastrointestinal perforation, bleeding, arterial thromboembolic events, infection, hypertension
VEGF Inhibitor	Aflibercept	VEGF-A, VEGF-B, PlGF	Eye stinging, eye inflammation, blurred vision, eye bleeding, intraocular infection, increased eye pressure, retinal detachment
VEGF Inhibitor	Ramucirumab	VEGF Receptor-2	Bleeding, gastrointestinal perforation, Wound healing complications, arterial thromboembolic events, hypertension, infusion-related reactions, proteinuria
PDGF Inhibitor	Imatinib	PDGF receptor signaling	Nausea, vomiting, myelosuppression
IL-23 Inhibitor	Guselkumab	p19 subunit of IL-23	Respiratory infection, headache, arthralgia, diarrhea, gastroenteritis, tinea infection, herpes simplex infection.
Anti-TNF-α Therapy	Infliximab	TNF-α	Infections, infusion reactions, autoimmune phenomena, headache, nausea, rash
Anti-TNF-α Therapy	Adalimumab	TNF-α	Infections, infusion reactions, autoimmune phenomena, headache, musculoskeletal pain
Anti-TNF-α Therapy	Golimumab	TNF-α	Infections, infusion reactions, autoimmune phenomena, tumor, cytopenia, allergy, headache, hypertension, rash
MMP Inhibitors	(Under study)	Matrix metalloproteinases	In early development (side effects not yet defined)
Endothelial Cell-Targeting Therapies	(Under study)	Endothelial cell signaling pathways	In preclinical/clinical validation (side effects not yet defined)
